# Sexual history taking: Doctors’ clinical decision-making in primary care in the North West province, South Africa

**DOI:** 10.4102/phcfm.v13i1.2985

**Published:** 2021-09-29

**Authors:** Deidré Pretorius, Ian D. Couper, Motlatso G. Mlambo

**Affiliations:** 1 Division of Family Medicine, School of Clinical Medicine, University of the Witwatersrand, Johannesburg, South Africa; 2 Ukwanda Centre for Rural Health, Faculty of Medicine and Health Sciences, Stellenbosch University, Stellenbosch, South Africa; 3 Department of Institutional Intelligence, University of South Africa, Pretoria, South Africa

**Keywords:** vignette, sexual dysfunction, clinical reasoning, decision-making, primary care, hypertension, diabetes

## Abstract

**Background:**

Clinical reasoning is an important aspect of making a diagnosis for providing patient care. Sexual dysfunction can be as a result of cardiovascular or neurological complications of patients with chronic illness, and if a patient does not raise a sexual challenge, then the doctor should know that there is a possibility that one exists and enquire.

**Aim:**

The aim of this research study was to assess doctors’ clinical decision-making process with regards to the risk of sexual dysfunction and management of patients with chronic illness in primary care facilities of the North West province based on two hypothetical patient scenarios.

**Setting:**

This research study was carried out in 10 primary care facilities in Dr Kenneth Kaunda health district, North West province, a rural health district.

**Methods:**

This vignette study using two hypothetical patient scenarios formed part of a broader grounded theory study to determine whether sexual dysfunction as comorbidity formed part of the doctors’ clinical reasoning and decision-making. After coding the answers, quantitative content analysis was performed. The questions and answers were then compared with standard answers of a reference group.

**Results:**

One of the doctors (5%) considered sexual dysfunction, but failed to follow through without considering further exploration, investigations or management. For the scenario of a female patient with diabetes, the reference group considered cervical health questions (*p* = 0.001) and compliance questions (*p* = 0.004) as standard enquiries, which the doctors from the North West province failed to consider. For the scenario of a male patient with hypertension and an ex-smoker, the reference group differed significantly by expecting screening for mental health and vision (both *p* = 0.001), as well as for HIV (*p* < 0.001). The participating doctors did not meet the expectations of the reference group.

**Conclusion:**

Good clinical reasoning and decision-making are not only based on knowledge, intuition and experience but also based on an awareness of human well-being as complex and multidimensional, to include sexual well-being.

## Background

Clinical decision-making based on the patient’s medical history is an important aspect of medical care provision and requires attentive engagement on the part of the practitioner.^1^ This is no less true when it comes to clinical reasoning in terms of sexual health, where taking a sexual history is arguably even more important because mostly there is very little that clinical examination or special investigations may add to the understanding of the problem. Various studies have found that doctors seldom take a sexual history and even less often screen for sexual dysfunction.^2,3,4,5,6^ Research studies have also shown that patients do not necessarily know that their illness or medication can sometimes cause sexual challenges; however, they are usually keen to discuss their sexual challenges with doctors.^5,7,8^

Admittedly, improved patient help-seeking behaviour can ease the exploration of sexual dysfunction. However, being a disease expert, doctors would anticipate and create the opportunities to talk about sexual challenges.^7^ The question is whether sexual dysfunction as a comorbidity features in the doctors’ clinical reasoning and decision-making? Atkinson et al.^9^ defined clinical reasoning as a thinking and decision-making process in professional practice, which is context dependent and guides practice actions. Case-specific knowledge, the skill to cognitively process the information, and reflective self-awareness are requirements for optimal clinical reasoning.^9^ Some educators add pattern recognition and a dose of intuition to the cognitive skills a doctor needs.^10,11^ Young et al.^12^ organised clinical reasoning in terms of acquired knowledge; interconnected and flexible knowledge organisation; coordinated and contextualised cognitive processing; and most importantly, metacognitive processing. Metacognitive processing includes reflexivity to improve judgement and performance.^12^ Dennick^11^ postulated that to consider a possible diagnosis, hypothetico-deductive reasoning must include background knowledge and experience to assimilate the inductive phase and reduce hypothesis formation from the imagination. We know that availability bias where the frequency of a condition is overestimated, as well as being so vested in a diagnosis, results in doctors not considering an alternative diagnosis, thus contributing to diagnostic errors.^13^ In this research study, the formation of a hypothesis of sexual dysfunction or considering sexual dysfunction as an alternative diagnosis based on knowledge and experience, or to include it as part of a hypothetical management plan, could suggest that sexual dysfunction was included in the cognitive processing of information and thus formed part of clinical reasoning and decision-making.

Vignette methodology has been used to assess cognitive processing in clinical reasoning and decision-making.^12^ Jiwa et al.^14^ used vignettes to compare clinical management decisions between general practitioners and diabetologists in Australia. In another study, vignette methodology was used to develop a successful tool to train health care workers in primary care to talk more frequently about sex in a consultation.^15^ Results from a vignette-based study in the United States and the United Kingdom revealed that patients’ age, race and gender, as well as doctors’ gender and experience, influenced clinical decision-making for diabetes.^16^ A vignette study on probable risks for sexually transmitted infections (STIs) and HIV suggested that stigmatisation influenced the risk perception of doctors.^17^ Although the focus of these studies was not sexual dysfunction, the factors influencing the reasoning and judgement were highlighted, and therefore, could be used to understand the reasoning that influences the risk assessment and management of sexual dysfunction presenting in chronic illnesses. Doctor-patient knowledge disparities with regards to the expression of sexuality in chronic disease or old age, personal perceptions and expectations based on previous encounters seem to influence doctor’s judgement when it comes to sexual health.^18,19,20,21,22^

The use of a vignette is a valid strategy to assess clinical judgement and decision-making around a diagnostic problem. Moreover, a vignette has good discriminative values, a good criterion and content validity, as well as consistently measure physician practice.^23,24^ If the vignette is well-designed with specific questions, the outcome in terms of judgement and decision-making is highly generalisable to everyday practice and decisions.^23^ It also measures practice more precisely than chart abstractions and is closer to assessments with standardised patients.^24^ The researcher decided to present doctors with two hypothetical vignette-based patient scenarios in order to assess whether they would consider the risk of sexual dysfunction and what role, if any, it would play in their management decision-making.

## Aim and objectives

The aim of this research study was to assess doctors’ clinical decision-making process with regards to the risk of sexual dysfunction and management of patients with chronic illness in primary care in the North West province based on two hypothetical patient scenarios by assessing the doctors’ diagnostic perception and management of two hypothetical case studies; to compare the scenario outcomes with a reference group outcome of the same scenarios, and to describe the clinical reasoning and decision-making process.

### Study design

This vignette study formed part of a broader grounded theory study,^25^ focussing on taking of history of sexual dysfunction in routine consultations. Part 1 of the study recorded and analysed 151 routine consultations with patients at risk of sexual dysfunction, to observe if and how sexual history taking presented in consultations and was reported in other articles. The second part of the study sought to determine whether the possibility of sexual dysfunction as a comorbidity formed part of the doctors’ clinical reasoning and decision-making process using a vignette method. It was also used to gauge the trustworthiness of the observation outcomes of the broader study.

### Setting

Dr Kenneth Kaunda Health District in the North West province was selected as a research site. At the time of the study, about 28 doctors were supporting nurse practitioners in 10 of the 36 primary care facilities located in this rural health district.

### Sampling strategy

A stepwise sampling approach was employed, involving recruitment of the clinics first, and then the doctors. The recruitment criteria included the clinics with a doctor to be consulting there at least once per week. Ten clinics along with 28 doctors working in these clinics across the health district were included. About 19 doctors consented to participate in the second part of the research study, which involved responding to questions on hypothetical patient scenarios.

### Vignette scenarios

The scenarios for the vignettes were created based on real data of two patients, in which the doctors had to consider the differential diagnosis, complications and management.

Scenario 1:

- A 48-year-old female patient with type 2 diabetes mellitus (DM) presents in your consulting room with complaints of vaginal burning and itchiness. She has no suprapubic pain and confirms a slight discharge. She has a history of non-infectious vulvovaginitis. Vital signs, random blood glucose level and haemoglobin A1c (HbA1c) are within the normal range.

Questions on Scenario 1 were as follows:

- What are the possible conditions that could be affecting this patient?
- What would you like to ask the patient to help her?
- How will you manage this patient based on the given information?

Scenario 2:

- A 45-year-old male patient with hypertension for the past 10 years presents for his annual check-up. He is at present on Atenolol (Tenormin) 100 mg daily and Enalapril 10 mg b.d. (twice a day) and hydrochlorothiazide (HCTZ) 12.5 mg. He is happy to inform you that he stopped smoking three months ago (he smoked 18 cigarettes per day since age 17). He complains of fatigue.

Questions on Scenario 2 were as follows:

- What are the possible complications will you explore?
- How will you manage this patient based on the given information?

### Pilot study

The two scenarios were piloted with 15 family physicians and family medicine registrars in the Ekurhuleni health district (a district in a different province from the study district), and no changes were suggested.

### Data collection

During data collection, both the researcher and the trained research assistant approached the doctors involved in the video recordings of consultations and briefed them on the second part of the study, namely, the hypothetical scenarios. The doctors who participated were asked to complete a demographic questionnaire and answer the questions on the two vignettes. Doctors completed this session in the presence of the researcher and could take as much time as needed to complete their responses before the answered questionnaires were collected. The doctors were blinded to the focus on sexual dysfunction and sexual history taking.

### Data analysis

**FIGURE 1:**
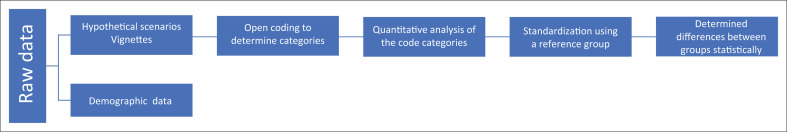
Data analysis for this study.

The data from the demographic questionnaires were captured in Microsoft Excel. The responses to the question(s) on the vignettes were imported into MaxQDA 2018 software and analysed. Raw data were coded using an open coding approach to determine categories emanating from data after which quantitative content analysis was carried out (Figure 1). Coding was reviewed by a panel of a public health specialist, a general practitioner and the two study supervisors. When the frequency of responses was known, a reference group consisting of three medical doctors (a trainer of final-year medical students and two working as generalists in the public sector), as well as one of the family physicians involved with training of undergraduate students, registrars and interns, were asked to standardise the answers (Figure 1). Their work experience varied between 3 years and 35 years after graduation. They were given the questions and answers the primary care doctors in North West province gave to the scenario questions and then requested to indicate how many junior doctors or interns out of a possible 10 would they expect to give the answers they considered a standard answer. Consensus was sought to a mean number of correct responses amongst the reference group, where possible, but if this was not possible, the mean of the suggested number of expected correct responses was calculated. Where the reference group agreed with the doctors in the North West province that it was an ideal answer, but differed from a clinical perspective on the feasibility, it was also documented. The frequency of qualitative answers to the questions in the vignettes from the doctors in the North West province, and those of the reference group were expressed as percentages. The level of agreement or differences between the doctor participants and the reference group was assessed using Fischer’s exact test at a 95% confidence level.

### Ethical considerations

The study was approved by the Human Research Ethics Committee (Medical) of the University of the Witwatersrand, Johannesburg (M160557). The Directorate Research, Monitoring & Evaluation of the Department of Health, North West province, South Africa, granted permission for proceeding with the research. The district and clinic managers also granted permission to carry out the research in their facilities. Clinics were not identified and only referred to as site numbers to ensure confidentiality of both setting and healthcare workers. Confidentiality was maintained as the questionnaires were numbered, and doctors completed them anonymously.

## Results

### Participant demographics

Six female and 13 male doctors participated in the research study, with the female doctors being much younger than their male counterparts (Table 1). The sample included two interns, three community service doctors and 14 medical officers. The male doctors were mostly career public medical officers with more experience (median for men 192 months vs. 36 months for women).

**TABLE 1:** Characteristics of doctors.

Characteristic	Male (*n* = 13)	Female (*n* = 6)
**Age**		
Range (years)	25–67	25–34
Mean (years)	36	29
Median (years)	34	29
**Marital status**		
Married		
*n*	6	4
%	46	67
Single		
*n*	7	2
%	54	33
**Level of education**		
MBChB only		
*n*	3	-
%	23	-
MBChB and additional diplomas		
*n*	7	3
%	54	50
MBChB and other degrees		
*n*	3	3
%	23	50
**Home language**		
Afrikaans		
*n*	4	3
%	31	50
English		
*n*	1	1
%	8	17
French		
*n*	4	-
%	31	-
Mandarin		
*n*	1	-
%	8	-
Sesotho		
*n*	1	-
%	8	-
Setswana		
*n*	1	2
%	8	33
Spanish		
*n*	1	-
%	8	-
**Work experience**		
Range (years)	2–43	2–7
Median (years)	16	3
Mean (years)	12	4

### Vignette results: Clinical reasoning for the two scenarios

#### Scenario 1

Scenario 1 was about a 48-year-old female patient with type II DM who presented with complaints of vaginal burning and itchiness. The results revealed that doctors were leaning towards a *Candida* infection, STI and vaginal atrophy as possible conditions affecting the hypothetical patient (Table 2). The reference group agreed on these as possible options; however, they considered menopause as the standard answer for this scenario. There was a statistical difference between the doctors’ responses and the responses of the reference group on the diagnosis of menopause (*p* = 0.025) (see Table 2).

**TABLE 2:** Hypothetical scenario 1: a 48-year-old female patient with diabetes.

Question	Responses	Doctor responses (%)	Responses expected by reference group (%)	*p*
What are the possible conditions that could be affecting this patient?	*Candida* infection	58	83	0.058
	STI or VDS	47	40	0.779
	Vaginal atrophy	32	33	1.000
	UTI	26	10	0.131
	Menopause	5	24	0.025
What would you like to ask her to help her?	Reproductive health questions	42	30	0.39
	Cervical health questions, including last cervical smear	11	60	0.001
	Sexual dysfunction	0	8	0.544
	Compliance (adherence to medication)	11	50	0.004
	Vaginal hygiene	21	0	0.009
How will you manage this patient based on the given information?	Offer cervical smear	16	78	< 0.001
	Do urine dipstick	7	53	< 0.001
	Teach wiping techniques	7	43	0.005
	Offer HIV test or VCT	11	90	< 0.001
	Prescribe anti-fungal treatment	63	90	0.028
	Suggest lubricants and/or topical oestrogen	5	0	0.322

STI, sexually transmitted infections; VDS, venereal disease; UTI, urinary tract infection; VCT, voluntary testing and counseling.

The questions the doctors would have preferred to ask the hypothetical patient focussed mainly on the attributes of the symptoms and past medical history. Two doctors (11%) wrote questions that could have elicited sexual dysfunction, namely:

‘How about sexual relationship with partner?’ (Dr 03, 35-year-old male doctor)

‘Pain and tenderness during intercourse?’ (Dr 25, 26-year-old female doctor)

**FIGURE 2:**
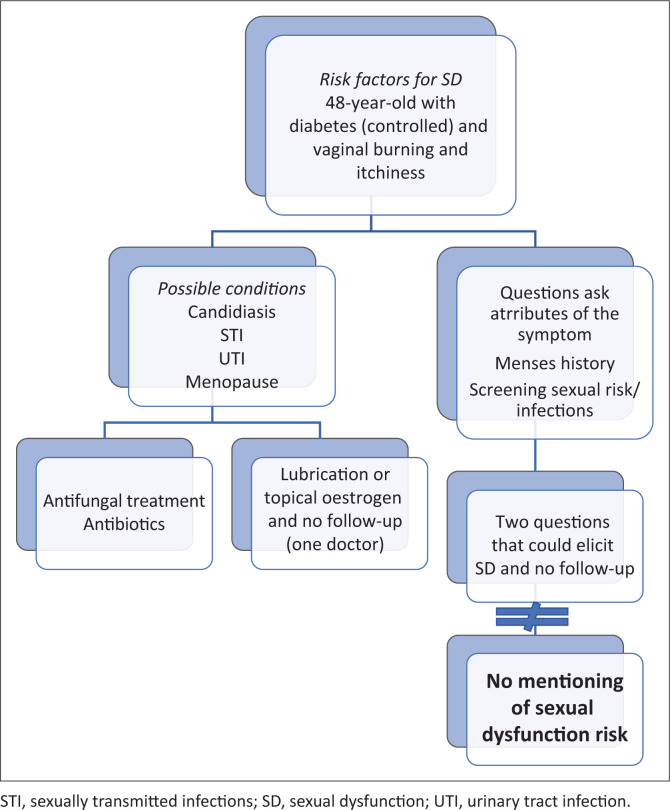
Clinical reasoning flow diagram of doctors for Scenario 1.

Sexual dysfunction specifically was never mentioned in the responses to the scenario, and there was no follow through on the abovementioned questions that could have led to eliciting it (Figure 2). Despite the reference group expecting that at least 8% of junior doctors would have enquired about sexual dysfunction, there was no statistically significant difference between the two groups. The reference group considered checking cervical health, including last cervical smear (*p* = 0.001), and checking compliance with medication (*p* = 0.004) as standard and appropriate enquiries that the doctors in the North West province failed to consider. These doctors, however, exceeded the reference group’s set standard on vaginal hygiene questions (*p* = 0.009).

Contrary to the expectations of the reference group, the participating doctors considered the following management options: offering cervical smear (*p* < 0.001), HIV test (*p* < 0.001), urine dipstick (*p* < 0.001), teaching of wiping techniques (*p* = 0.005) and prescribing antifungal treatment (*p* = 0.028). The reference group considered lubricants or topical oestrogen as a standard answer; however, they did not consider it as a reasonable answer for the management because of the lack of availability of these in the public health sector.

Figure 2 reflects the flow of responses to Scenario 1, showing that the clinical reasoning did not include sexual dysfunction for most of the doctors.

#### Scenario 2

Scenario 2 was about a 45-year-old male patient with hypertension, who presented for his annual check-up and complained about fatigue. He had stopped smoking 3 months ago. As shown in Table 3, the doctors prioritised cardiac failure as a possible complication and focus of management (27% and 18% of the responses, respectively), and the reference group considered it as a standard answer.

**TABLE 3:** Scenario 2: A 45-year-old male patient with hypertension and smoking history presenting with fatigue.

Question	Responses	Doctor responses (%)	Responses expected by reference group (%)	*p*
What are the possible complications will you explore?	CCF	58	51	0.591
	Bradycardia	5	15	0.411
	Medication side effects	21	18	0.734
	Psychosocial complications	11	55	0.002
	Renal complications	53	50	1.000
	Respiratory complications	42	68	0.090
	Sex life complications	5	3	0.544
How will you manage this patient based on the given information?	ECG	63	38	0.094
	Blood tests	42	58	0.034
	Smoking cessation	53	0	< 0.001
	Review medication	26	25	1.000
	Mental health	16	63	0.001
	HIV test	11	85	< 0.001
	Visual screening	16	78	< 0.001
	Compliance or adherence	5	50	0.001
	Lifestyle	16	35	0.218
	Further history	11	53	0.002
	Refer	5	0	0.322
	Lung function test	26	5	0.030
	Radiology	11	0	0.100

CCF, congestive heart failure; ECG, electrocardiogram.

The reference group, however, expected the participating doctors to mention psychosocial and respiratory complications, which they failed to do. The reference group differed significantly by expecting the participating doctors to screen for mental health and vision (both *p* = 0.001), as well as for HIV (*p* < 0.001). The participating doctors wanted to discuss smoking cessation with the hypothetical patient, and the reference group considered it as inappropriate (*p* < 0.001).

**FIGURE 3:**
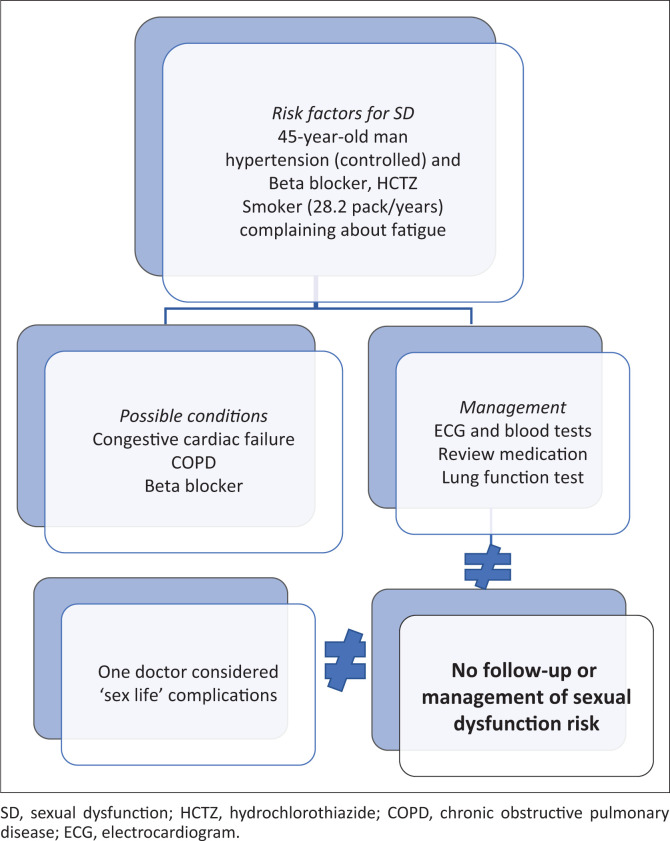
Clinical reasoning flow diagram, Scenario 2.

One of the male doctors (a 35-year-old) mentioned ‘sex life’ as a possible complication; however, it was not followed through in management (Figure 3). As summarised in Figure 3, the flowchart of clinical reasoning on this hypothetical scenario has been prepared; sexual dysfunction specifically was not considered.

## Discussion

The researcher used questions on the diagnosis and management of hypothetical patient scenarios to assess doctors’ clinical reasoning and decision-making regarding the risk of sexual dysfunction in patients with chronic illness. Despite 14 out of 19 (74%) primary care doctors in this sample being career and senior medical officers, their overall clinical reasoning assessed with hypothetical patient scenarios was below the standard that the reference group expected from an intern or junior doctor. Although the reference group agreed that some of the responses were clinically good, they were unpractical management options, such as ECGs, lung function tests and lubricants not readily available in the primary care clinics. As the answers to the scenarios did not reflect that the doctors considered referring the patients for such a service, their answers were interpreted to indicate what they wanted to do within the context they worked at that time, namely, a primary healthcare clinic that could not provide many of the investigated or treatment modalities listed. It was also clear that the doctors did not comply with guidelines on the offering of HIV tests,^26^ cervical smear,^27^ and mental health screening^28^ for patients with chronic disease. Both the hypothetical patients were at risk of sexual dysfunction, but only one doctor considered exploring sexual functioning (the doctor did not specify the type of dysfunction, calling it ‘sex life’) in response to these scenarios, and none included it as a management option. Although sexual functioning is important for the patient’s well-being, it did not seem to be part of the overall clinical reasoning of the primary care doctors in North West province. It is thus unlikely that they would have explored it in real patients. According to Kingsberg,^29^ sexual health seldom has a high priority in medical education. The neglect of sexual functioning is likely starting in undergraduate training where students memorise and use symptom lists, algorithms and guidelines, in which reproductive health and sexual risk behaviour feature, but not necessarily sexual functioning. Doctors are trained with a focus on disease and not on health; they are taught to look for pathology rather than focusing on and supporting well-being.

If we consider clinical reasoning as the fault line for not thinking of sexual dysfunction in this study, the question is what doctors already know, as knowledge is the starting point for clinical reasoning. They knew that both hypertension and diabetes have target organ complications - sometimes also referred to as the ‘vascular tree’ that accelerates the decrease of kidney function, retinopathy and cerebral functioning or disease. It would be the responsibility of a doctor to set therapeutic goals and manage cardiovascular risks aggressively, certainly in the initial stages; however, long-term management of the patients must be based on a more holistic understanding.^30^ Exploring decision-making or judgement analysis in terms of diabetes and hypertension management, it was clear that decision-making was most of the time based on the management of control targets and/or the risk-benefit ratios.^14,31,32,33,34,35,36,37^ Based on a knowledge base, they could thus organise their knowledge to consider examples of typical complications of diseases that the symptoms represented.

In Scenario 1, lubrication was considered; however, the fact that sexual desire and orgasmic dysfunction can coexist was not. The patient in Scenario 2 did not only have hypertension and was under medication with a risk of erectile dysfunction; however, the previous history of smoking had also increased the risk of sexual dysfunction.^38^ It thus seems that the doctors, in this study, may lack the knowledge on sexual dysfunction, which is in line with other research findings.^39^ However, this also raised a red flag in terms of cognitive processing where clinical information must be used to generate a test hypothesis.^12^ It seems that the doctors answered the questions rapidly and intuitively, mainly concentrating on the initial diagnosis and presenting complaint. This is also known as automated reasoning and is common in decision-making.^40^

If there was deliberate or deep and analytical thinking^41^ about the symptoms the hypothetical patients presented with, they might have considered other risks, such as the microvascular complications that also occur in the genitalia, for example, erectile failure in men, which is common knowledge but still not optimally addressed in primary care,^42^ and, perhaps less known, clitoral vascular resistance in women with insulin resistance.^43^ They considered cardiovascular disease; however, did not consider that erectile dysfunction is also an early biomarker of cardiovascular disease, and thus, it is a good clinical practice to screen for it.^44^ Chronic diseases are known for comorbidities. Therefore, from a clinical and pharmacological point of view, there is an expectation that if the doctor knows that a patient lives with certain diseases and uses certain medications, he or she would screen for sexual dysfunction.^45,46^ Medications, such as antihypertensives, diuretics, antidepressants, antipsychotics, mood stabilisers and others, are known for side effects of sexual dysfunction.^47^

Lifestyle issues, such as past and current smoking habits, excessive use of caffeine use and lack of physical activity, also increase the chances of sexual dysfunction and need to be considered in the management of patients with chronic disease.^38^ This type of analytical reasoning was not evident in the answers generated in this study. Mamede et al.^48^ found that analytical reasoning and medical experience were proportionately and inversely associated with each other, which could explain the lack of analytical thinking in this sample of male doctors with a median experience of 16 years. There is, however, no explanation for the less experienced doctors not to exclude analytical thinking.

If there was a deliberate or deep and analytical thinking^41^ about the symptoms presented by the hypothetical patients, they would have considered other risks, such as microvascular complications, to also occur in the genitalia, for example, erectile failure in men, which is common knowledge but still not optimally addressed in primary care,^42^ and, perhaps less known, clitoral vascular resistance in women with insulin resistance.^43^ They considered cardiovascular disease; however, erectile dysfunction was also not an early biomarker of cardiovascular disease, and thus, good clinical practice to screen for it.^44^

Patients of all ages experience sexual challenges, and patients with diabetes and hypertension experience an even more significant burden of disease.^49,50,51,52,53,54,55^ As doctors are expected to serve the best overall benefit or the best interest of the patient, many researchers have concluded that screening for sexual dysfunction must be a clinical priority when dealing with patients living with chronic disease.^2,56^ Best interest should also imply giving attention to holistic well-being, although it has been shown that patients’ preferences and subjective illness experiences are often not a consideration in clinical judgement or decision-making.^19,57,58,59^ It seems sexual history taking in practice is not core to the decision-making process in the management of patients with chronic disease despite being a theoretical and health priority.

It was evident that doctors focussed on an initial diagnosis and presenting complaint. Gay et al.^60^ emphasised that managing the initial diagnosis is not good enough as clinical reasoning extends beyond it in the management of patients. The results of this study also did not suggest any form of reflexivity, also known as meta-reasoning^48^ to check errors in their reasoning or to reconsider the diagnosis or management. The dual-process theory does not negate the importance of intuition and pattern recognition acquired through experience in clinical reasoning and decision-making but also emphasises the meta-cognitive control to reconsider and reduce errors applying analytical reasoning.^41^ The results of this study lacked the evidence of metacognitive and analytical reasoning.

Where is the gap? Do doctors lack only the knowledge of sexual health, more specifically knowledge of sexual dysfunction? Or is it a deeper problem at a cognitive processing and an awareness level? Both can be addressed by training doctors during undergraduate years. Various studies have shown how training doctors has improved the knowledge gap in and practice of sexual history taking.^61,62,63,64,65,66,67^ Most importantly, doctors must be taught to enquire about their own beliefs and assumptions, which requires a humble openness to generate alternative explanations for what seems to be obvious.^48^

## Conclusion

Good clinical reasoning and decision-making are based on not only knowledge, intuition and experience but also an awareness of human well-being as complex and multidimensional to include sexual well-being. Therefore, the main focus should be on clinical reasoning whilst training of doctors during undergraduate years, a sexual health curriculum that cuts across specialist disciplines and development of skills to communicate matters of sexual functioning.
